# *Plasmodium knowlesi* infecting humans in Southeast Asia: What’s next?

**DOI:** 10.1371/journal.pntd.0008900

**Published:** 2020-12-31

**Authors:** Nantha Kumar Jeyaprakasam, Jonathan Wee Kent Liew, Van Lun Low, Wan-Yusoff Wan-Sulaiman, Indra Vythilingam

**Affiliations:** 1 Department of Parasitology, Faculty of Medicine, University of Malaya, Kuala Lumpur, Malaysia; 2 Tropical Infectious Diseases Research and Education Centre (TIDREC), University of Malaya, Kuala Lumpur, Malaysia; Johns Hopkins Bloomberg School of Public Health, UNITED STATES

## Abstract

*Plasmodium knowlesi*, a simian malaria parasite, has been in the limelight since a large focus of human *P*. *knowlesi* infection was reported from Sarawak (Malaysian Borneo) in 2004. Although this infection is transmitted across Southeast Asia, the largest number of cases has been reported from Malaysia. The increasing number of knowlesi malaria cases has been attributed to the use of molecular tools for detection, but environmental changes including deforestation likely play a major role by increasing human exposure to vector mosquitoes, which coexist with the macaque host. In addition, with the reduction in human malaria transmission in Southeast Asia, it is possible that human populations are at a greater risk of *P*. *knowlesi* infection due to diminishing cross-species immunity. Furthermore, the possibility of increasing exposure of humans to other simian *Plasmodium* parasites such as *Plasmodium cynomolgi* and *Plasmodium inui* should not be ignored. We here review the current status of these parasites in humans, macaques, and mosquitoes to support necessary reorientation of malaria control and elimination in the affected areas.

## Introduction

For many years, it has been accepted that only 4 species of *Plasmodium* namely *Plasmodium falciparum*, *Plasmodium vivax*, *Plasmodium malariae*, and *Plasmodium ovale* cause natural malaria infection in humans. However, this assumption was challenged when the first case of naturally acquired *Plasmodium knowlesi* infection was reported in an American army surveyor in the state of Pahang, Malaysia in 1965 [1].

The long-tailed macaque (*Macaca fascicularis*) was identified as a natural host of *P*. *knowlesi*, *Plasmodium fieldi*, *Plasmodium cynomolgi*, *Plasmodium coatneyi*, and *Plasmodium inui*, among others [2,3]. However, no mosquitoes were found to harbor sporozoites of these *Plasmodium* species in the area where the first case was reported. In contrast, *Anopheles hackeri* was incriminated as the vector of *P*. *knowlesi* in the state of Selangor and found to be attracted to nonhuman primates rather than humans, biting especially at canopy level [4]. Thus, it was postulated that human infection with simian malaria parasites is a rare event [5].

However, this scenario was greatly challenged when a large focus of human *P*. *knowlesi* infection was reported among the local population in Sarawak, Malaysia in 2004 [6]. It is believed that *P*. *knowlesi* infections in humans had been there much earlier but were only detected when molecular tools were adopted [7]. Under experimental conditions, both *P*. *inui* [8] and *P*. *cynomolgi* [9,10] can infect humans through mosquito bites, while natural *P*. *cynomolgi* infection had been reported recently [11–15]. On this background, we here review the current occurrence of *P*. *knowlesi* in human, mosquito, and macaque hosts, examining also the possible emergence of other simian *Plasmodium* species as zoonoses in Southeast Asia.

## Methodology

A literature search was carried out using PubMed/Web of Science, Google Scholar, and other sites to find relevant materials related to simian malaria in humans, macaques, and vectors. The following search terms were used singly or in combination: simian malaria, primate malaria, zoonotic malaria, non-human primate, *Anopheles*, malaria vector, macaques, and monkeys. The most relevant publications related to the current topic were selected. All relevant early to present publications were included. Since the focus of the review article is on Southeast Asia, other simian *Plasmodium* species infecting humans in other parts of the world are not discussed in detail.

## Epidemiology

Human infection by *P*. *knowlesi* had been reported from all Southeast Asia countries except Timor Leste [16] (Table 1).

**Table 1:** Knowlesi malaria cases in SEA based on the cumulative cases confirmed by PCR and/or sequencing and reported in peer-reviewed published articles.

Country	Year	No. of *P*. *knowlesi* cases	References
Brunei	2007–2017	73	[17]
Cambodia	2007–2010	2	[18]
Indonesia	2008–2015	418	[19–24]
Laos	2010–2016	10	[25,26]
Malaysia	2010–2018	18,687	[27]
Myanmar	2008–2013	49	[28–30]
Philippines	2006	5	[31]
Singapore	2007–2008	6	[32,33]
Thailand	2000–2018	44	[29,34–40]
Vietnam	2004–2010	38	[26,41,42]

PCR, polymerase chain reaction; SEA, Southeast Asia.

### Knowlesi malaria in Malaysia

Malaysia, notably Sabah and Sarawak, reported the highest numbers of *P*. *knowlesi* cases in Southeast Asia [43] following the drastic reduction of malaria cases caused by the human malaria parasites. Knowlesi malaria now accounts for all local cases reported [44].

In Sabah, it was demonstrated that knowlesi malaria spread gradually from areas with no transmission of human malaria to other areas, as human malaria cases were reduced [45]. Most cases were found in the Southwest interior region, gradually spreading to the West Coast and then on to the northern area and finally to the East Coast where *P*. *vivax* was still present in substantial numbers [45]. In Sarawak, there were a total of 9,364 *P*. *knowlesi* cases from 1992 to 2014 [46]. From 73 cases in 1992, the number increased dramatically to 1,095 in 2014, far exceeding the incidence in Peninsular Malaysia [46]. Environmental changes especially associated with deforestation and land exploration bring human population in close contact with *Anopheles* mosquito vectors over time, which inevitably increases the risk [47,48].

### Knowlesi malaria in other Southeast Asian countries

Besides Malaysia, large numbers of knowlesi malaria cases were recorded in neighboring countries, Brunei, Indonesia, and Thailand (Table 1). The low reported incidence of knowlesi malaria in other Southeast Asia countries might be due to misdiagnosis through microscopy and a scarcity of specific studies [49]. Thus, the knowlesi malaria cases reported from these countries may be just the tip of the iceberg. This underlines the importance of multinational collaboration in reducing or eliminating knowlesi malaria in future.

### Knowlesi malaria among travelers

**Fig 1:**
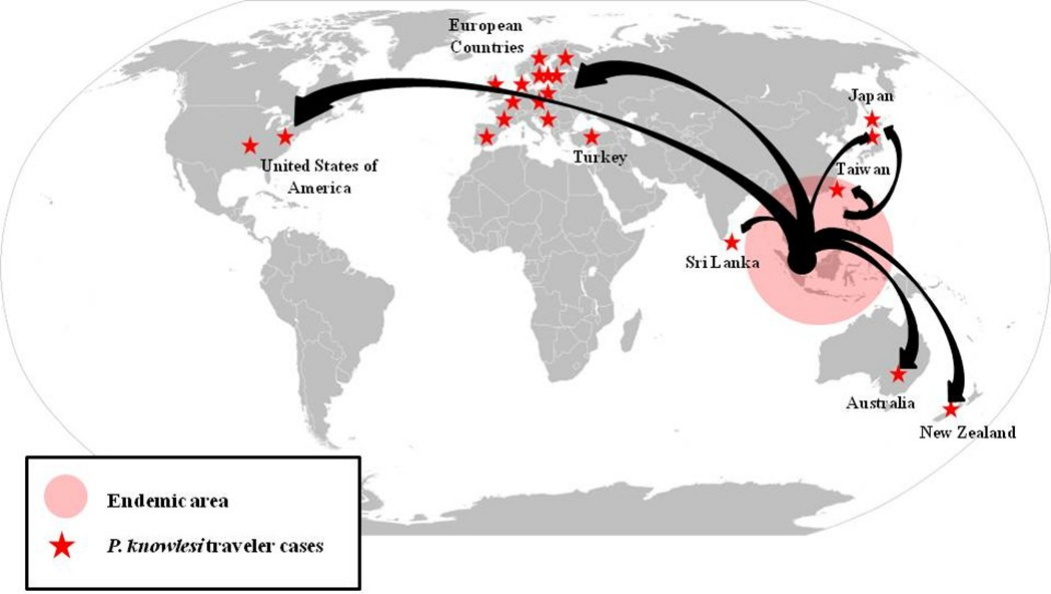
*Plasmodium knowlesi* exported from SEA to other areas across the world. SEA, Southeast Asia.

There are also increasing numbers of cases of knowlesi malaria imported from Southeast Asia to Europe, Asia, America, and Oceania (Fig 1) [50,51]. The countries outside Southeast Asia which had reported imported *P*. *knowlesi* malaria in international travelers are listed in S1 Table.

### Other simian malarias in humans

The first reported case of a natural transmission of *P*. *cynomolgi* was from Hulu Terengganu, the east coast of Peninsular Malaysia in 2011 [11]. Following that, natural infections of *P*. *cynomolgi* in humans became increasingly evident in Sarawak [13], northern Sabah [12], and western Cambodia [14]. A *P*. *cynomolgi* case was reported in a tourist from Denmark who had visited Peninsular Malaysia and Thailand in 2018 [15].

In addition to human *Plasmodium* species, more infections of *P*. *knowlesi*, *P*. *cynomolgi*, *P*. *coatneyi*, and *P*. *inui* were detected in Malaysian samples through a PCR approach, some of which comprised mixed infections of *P*. *knowlesi* + *P*. *cynomolgi* and *P*. *knowlesi* + *P*. *coatneyi* [52]. These were detected from hospital samples as well as from community surveys [52] and highlight the importance of using diagnostic tests specific for these species.

## Macaque hosts

The main hosts of *P*. *knowlesi* are the long-tailed macaques (*M*. *fascicularis*), pig-tailed macaques (*Macaca nemestrina*), and banded leaf monkeys (*Presbytis melalophos*) [53]. The distribution and prevalence of simian *Plasmodium* in wild macaques in Southeast Asia are listed in Table 2. The compiled data from year 2004 until 2017 show that Malaysian Borneo has the highest prevalence of simian malaria in their wild macaques followed by Cambodia, Singapore, and Indonesia. In Malaysia, *M*. *fascicularis* has the highest prevalence of simian malaria. In Peninsular Malaysia, this species was positive for simian malaria in forested areas, but not in urban areas [54]. Overall, *P*. *knowlesi* and *P*. *inui* infections have similar rates in Peninsular Malaysia, followed by *P*. *cynomolgi* [54]. In contrast, in Sabah, *P*. *inui* infection is the most prevalent infection in long-tailed macaques, followed by *P*. *knowlesi* and *P*. *cynomolgi* with similar rate [48], while in Sarawak, *P*. *knowlesi* and *P*. *inui* have similarly high prevalence, followed by *P*. *coatneyi* and *P*. *cynomolgi* [55]. Most of the malaria–positive macaques in Peninsular Malaysia and Sarawak have mixed infections [55,56]. Besides, nonhuman primates in Southeast Asia harbor other simian *Plasmodium* parasites namely *Plasmodium eylesi*, *P*. *fieldi*, *Plasmodium fragile*, *Plasmodium hylobati*, *Plasmodium jefferyi*, *Plasmodium pitheci*, *Plasmodium simiovale*, *Plasmodium silvaticum*, and *Plasmodium youngi* [54]. Although these have not shown infection in humans, further studies are needed to determine if any of these species can cause human infections.

**Table 2:** Simian *Plasmodium* parasites in wild macaques in SEA.

Country	Location	Years	Type of macaques studied	Number of positive /number of samples tested (percentage positive)	Percentage of multiple infection among positive samples (%)	*P*. *knowlesi* (%)	*P*. *cynomolgi* (%)	*P*. *inui* (%)	*P*. *fieldi* (%)	*P*. *coatneyi* (%)	Unknown *Plasmodium* sp.	References
Cambodia	Vanny	2011	*M*. *fascicularis*	44/54 (81.5%)	27.3	-	50.0	22.2	1.9	29.6	-	[57]
Indonesia	Bintan Island	2007	*M*. *fascicularis*	16/20 (80.0%)	25.0	-	65.0	25.0	10.0	5.0	-	[57]
	Southern Sumatra	2010	*M*. *fascicularis*	49/50 (98.0%)	18.4	-	96.0	20.0	-	-	-	[57]
	Sumatra	Not stated	*M*. *fascicularis*	30/60 (50.0%)	NA	NA	NA	48.3	NA	NA	3.3	[58]
	West Java	Not stated	*M*. *nemestrina*	4/4 (100.0%)	NA	NA	NA	NA	NA	NA	NA	[58]
Laos	Guidong	2013	*M*. *fascicularis*	30/44 (68.2%)	3.3	2.3	63.6	-	2.3	-	-	[57]
Malaysia	Hulu Selangor	2014	*M*. *fascicularis*	35/70 (50.0%)	74.3	30.0	25.7	32.9	1.4	22.9	-	[56]
	Peninsular Malaysia	2015	*M*. *fascicularis*	NA	NA	34.3	27.9	33.2	27.6	16.6	-	[59]
	Kapit, Sarawak	2004–2008	*M*. *fascicularis*	80/82 (97.6%)	92.5	86.6	63.4	84.1	4.9	76.8	-	[55]
	Kapit, Sarawak	2004–2008	*M*. *nemestrina*	21/26 (80.8%)	66.7	50.0	34.6	76.9	-	34.6	-	[55]
	Sepilok, Sabah	2010–2011	*M*. *fascicularis*	26/26 (100%)	NA	15.4	11.5	30.8	3.8	3.8	3.8	[48]
	Sepilok, Sabah	2010–2011	*M*. *nemestrina*	15/15 (100%)	NA	13.3	6.7	60.0	20.0	6.7	6.7	[48]
Philippines	Montible subcolony and Iwahig Penal Colony, Palawan	1971–1972	*Macaca irus*	11/20 (55.0%)	27.3	0.0	9.1	9.1	0.0	0.0	54.5	[60]
	Batangas, Southwestern Luzon	2012	*M*. *fascicularis*	3/28 (10.7%)	33.3	-	10.7	-	-	3.6	-	[57]
	Zamboanga, Western Mindanao	2012	*M*. *fascicularis*	4/40 (10.0%)	-	-	2.5	5.0	-	2.5	-	[57]
	National Wildlife Rescue and Research Center, Manila	2017	Captive macaques	0/30 (0%)	-	-	-	-	-	-	-	[61]
	Puerto Princesa Subterranean River National Park, Palawan	2017	*M*. *fascicularis*	40/40 (100%)	90.0	45.0	57.5	92.5	90.0	47.5	-	[61]
	Palawan Wildlife Rescue and Conservation Center, Palawan	2017	*M*. *fascicularis*	5/25 (20.0%)	60.0	-	-	20.0	12.0	-	-	[61]
Singapore	Singapore	2007	*M*. *fascicularis*	31/40 (77.5%)	16.1	-	65.0	12.5	10.0	-	-	[57]
	Singapore	2007–2011	*M*. *fascicularis*	66/92 (71.7%)	42.4	48.9	43.5	1.1	12.0	2.2	-	[62]
	Singapore	2008–2011	Peri-domestic macaque	0/65 (0%)	-	-	-	-	-	-	-	[62]
Thailand	Ranong Province	2006	*M*. *fascicularis*	5/21 (23.8%)	20.0	-	-	23.8	-	4.8	-	[63]
	Prachuab Khirikhan Province	2006	Peri-domestic macaque	0/78 (0%)	-	-	-	-	-	-	-	[63]
	Southern Thailand	2008–2009	*M*. *fascicularis*	12/195 (6.2%)	16.6	0.5	0.5	3.6	-	1.5	0.5	[64]
	Southern Thailand	2008–2009	*M*. *nemestrina*	90/449 (20.0%)	11.1	1.1	1.1	14.0	0.2	0.4	5.1	[64]

SEA, Southeast Asia.

*P*. *knowlesi* has not been detected in wild macaques in Cambodia and Indonesia even though indigenous cases of knowlesi malaria in humans have been reported there [57,58]. Moreover, *P*. *knowlesi* is not the most prevalent malaria parasite found in macaques in each region/country in Southeast Asia except for Malaysian Borneo. The most prevalent simian malaria parasites in the other countries is either *P*. *inui* or *P*. *cynomolgi*. Both can reach more than 70% prevalence in some regions. Based on studies done thus far (Table 2), *P*. *inui* has the widest geographical distribution in Southeast Asia followed by *P*. *cynomolgi*. This is expected since these 2 parasites can infect a wide range of Old and New World monkeys (naturally or experimentally). Besides, *P*. *inui* is the widest-ranging parasite, likely due to its high chronicity in the hosts [65], followed by *P*. *cynomolgi*. It is also noteworthy that there is a generally high multispecies infection rate among the wild macaques. This leads to a bigger reservoir and higher risks of zoonotic transmission of *P*. *inui* and *P*. *cynomolgi* to humans.

## Vectors

### Distribution of *P*. *knowlesi* vectors

Most of the vector studies have been done in Malaysia [16] followed by Vietnam [42,66]. Studies in Malaysia first identified *An*. *hackeri* as the vector of *P*. *knowlesi* [4]. However, this species is so highly zoophilic that it has been considered impossible that it could infect humans [5]. After the recognition of the importance of *P*. *knowlesi* malaria in humans in Sarawak, vector studies were conducted in different ecological niches. In Kapit district, *Anopheles latens* was identified as the main natural vector of *P*. *knowlesi* for both macaques and humans [67], while in Sabah, *Anopheles balabacensis* was incriminated [68,69]. In both states, these 2 species, belonging to the Leucosphyrus group, are also vectors of human malaria [16].

In Pahang, Peninsular Malaysia, *Anopheles cracens*, a more anthropophilic member of the Leucosphyrus group was identified as the vector of *P*. *knowlesi* [70], while in Selangor, *An*. *hackeri* and *Anopheles introlatus* were found to be the vectors [4,71]. It seems that different geographical locations have a different predominant species of Leucosphyrus group. Within the same area, different ecological conditions play an important role. In Kapit, Sarawak *An*. *latens* and *Anopheles watsonii* were predominant in forested ecotypes, while in the farm areas, *Anopheles donaldi* and *An*. *latens* were the main species [67]. However, dissection and further examination of those mosquitoes revealed only *An*. *latens* to be positive with sporozoites and to be attracted to both humans and macaques. Thus, *An*. *latens* had been incriminated as the main vector for *P*. *knowlesi* in Kapit, Sarawak. The role of ecology was also observed by Wharton and Eyles, who incriminated *An*. *hackeri* as a vector for *P*. *knowlesi* at the coastal region of Selangor [4].

Other species of *Anopheles* like *An*. *watsonii* and *An*. *donaldi* found in sympatry with *An*. *latens* in Kapit were positive for oocysts with PCR–positive for the genus *Plasmodium*, but species identification by using primers for simian and human malaria parasites was not successful [67]. *Anopheles kochi* in Kuala Lipis, Pahang was found in considerable numbers in monkey baited traps but was not positive for oocyst or sporozoites by dissection [70]. A recent study by Hawkes and colleagues in Sabah found a non-Leucosphyrus group species, *An*. *donaldi*, to be positive for DNA of *P*. *knowlesi*, *P*. *cynomolgi*, and an unknown *Plasmodium* species [72]. Unfortunately, in this study, the whole mosquito was processed, so it is not known whether oocysts or sporozoites were present, and therefore, whether these mosquitoes were vectors.

In the forest and forest fringes of Khanh Phu commune, Vietnam, *Anopheles dirus*, the main vector of human malaria in this region [73], was identified as the primary vector of *P*. *knowlesi* [66]. It was the only *Anopheles* species found with sporozoites, and the highest number infected was detected in the forest compared to the forest fringes. *P*. *knowlesi* was the second most common *Plasmodium* species identified in the vectors using nested PCR assay followed by other simian plasmodia [66]. *P*. *knowlesi* was detected in the salivary glands of *An*. *dirus* as was the case in the previous study by Marchand and colleagues in the same locality [42].

In the other countries of the Mekong region (Cambodia, Laos, Myanmar, and Thailand), *An*. *dirus* is the main vector for human malaria besides *Anopheles minimus* [73]. Thus, based on results from Vietnam, it can be extrapolated that *An*. *dirus* could play a role in the transmission of knowlesi malaria in that region. In Palawan, Phillipines, Tsukamoto and colleagues demonstrated the presence of *Plasmodium* oocysts and sporozoites in *An*. *balabacensis* in an area where macaques were present. At that time, molecular tools were not available, so they carried out experiments to expose *An*. *balabacensis*, also a member of the Leucosphyrus group, to *Plasmodium*-positive macaques and showed that *An*. *balabacensis* could develop sporozoites [60]. In conclusion, it can be stated that, at present, only the Leucosphyrus group of *Anopheles* are confirmed vectors for *P*. *knowlesi* in Southeast Asia.

### Bionomics of the vectors

With extensive deforestation and land exploration in Malaysia over the past decades, there have been changes in the bionomics of the mosquitoes. Deforestation has caused the migration of the long-tailed macaques from forested area to farms and semi-urban areas where they usually scavenge for food. This may have triggered mosquitoes to follow the host and adapt to forest fringes and farm areas [16]. Fruit orchards that were propagated in those areas were ideal sites for the *Anopheles* vectors [74].

Most of these vectors tend to bite humans early, between 1900 and 2100 hours, but this varies with the geographical locations and the mosquitoes species [16,67–71]. *An*. *latens* prefers to bite macaques at 3 to 6 m above ground. It was demonstrated that the periodicity of the gametocyte stage in the macaques’ host coincides with the biting times of the vector mosquito [75]. This raises the possibility that these vector mosquitoes become infected by biting macaques late at night and transmitting the infection to humans by biting them in the early part of the night, when they are not protected.

### Other simian malaria parasites in vectors

Many studies have investigated the presence of other simian malaria parasites in salivary glands and oocysts from *Anopheles* mosquitoes using PCR. Based on the compiled data (Table 3), *P*. *cynomolgi* and *P*. *inui* were more prevalent in the vectors compared to *P*. *knowlesi*. These parasites were present as monoinfections and multiple infections. This observation is in parallel with the prevalence of simian plasmodia recovered from macaques [55,56,58]. The overlapping distribution of the vectors and the macaques in forested areas might explain the high prevalence of *P*. *cynomolgi* and *P*. *inui* in both the macaques and in the *Anopheles* mosquitoes. *Anopheles* mosquitoes identified as natural vectors for simian plasmodia in Southeast Asia are shown in Table 4. There are studies showing coinfections of these simian malaria parasites with human *Plasmodium* in mosquitoes [42,66,76] indicating possible simultaneous transmission.

**Table 3:** Simian *Plasmodium* parasites in *Anopheles* Leucosphyrus group in SEA.

*Plasmodium* species	Number of positive samples in *An*. *balabacensis* in Kudat and Pulau Banggi, Sabah, Malaysia [68]		Number of positive samples in *An*. *balabacensis* in Kudat and Ranau, Sabah, Malaysia [69,72,77]	Number of positive samples in *An*. *latens* in Kapit, Sarawak, Malaysia [78]		Number of positive samples in *An*. *cracens* in Kuala Lipis, Pahang, Malaysia [70]	Number of positive samples in *An*. *dirus* in Khanh Phu, Vietnam [66]	Number of positive samples in *An*. *balabacensis* in Palawan Island, Philippines [60]	
	Midgut	Salivary gland	Whole mosquito	Midgut	Salivary gland	Salivary gland	Salivary gland	Midgut	Salivary gland
Pk	-	1	1	-	5	3	4 (7*)	-	-
Pcy	7	6	5	-	-	-	6 (3*)	-	-
Pin	5	5	12	-	4	-	5 (1’) (3*) (1#)	-	-
Pfi	-	-	-	-	-	-	-	-	-
Pct	-	-	3	-	1	-	7	-	-
Pk + Pin	2	2	1	-	-	1	2 (2*)	-	-
Pk + Pcy	1	2	1	-	-	-	1*	-	-
Pcy + Pin	8	4	2	-	-	-	-	-	-
Pct + Pin	-	-	-	-	-	-	1*	-	-
Pfi + Pcy	-	-	1	-	-	-	-	-	-
Pin + Pfi	-	-	-	-	1	-	-	-	-
Pin + Pct	-	-	-	-	1	-	-	-	-
Pk + Pcy + Pin	-	4	-	-	-	-	-	-	-
Pk + Pct + Pin	-	-	-	-	-	-	1	-	-
Pct + Pcy + Pin	-	-	-	-	-	-	1*	-	-
Pk + Pct + Pcy + Pin	-	1	-	-	-	-	-	-	-
Not identified	8	2	-	5	-	-	-	7	3

Pct, *Plasmodium coatneyi*; Pcy, *Plasmodium cynomolgi*; Pfi, *Plasmodium fieldi*; Pin, *Plasmodium* inui; Pk, *Plasmodium knowlesi*; SEA, Southeast Asia.

(‘) Mixed with *P*. *falciparum*;

(*) Mixed with *P*. *vivax*;

(^#^) Mixed with *P*. *faciparum* and *P*. *vivax*.

**Table 4:** Natural vectors of common simian *Plasmodium* in SEA and their distribution.

Parasite	Natural vector	Place and method of incrimination	Distribution of the natural vector in SEA
*P*. *coatneyi*	*An*. *balabacensis*	Detection of parasite DNA in sporozoite-infected salivary gland. Sabah, Malaysian Borneo [68]	Brunei, Indonesia, Malaysian Borneo, and Philippines [79]
	*An*. *dirus*	Detection of parasite DNA in sporozoite-infected salivary glands. Vietnam [66]	Cambodia, Lao PDR, Thailand, and Vietnam [73]
	*An*. *hackeri*	Inoculation of sporozoites into rhesus macaque. Peninsular Malaysia [80]	Malaysian Borneo, Peninsular Malaysia, Philippines, and Thailand [73,79]
*P*. *cynomolgi* (including the variants *cyclopis* and *ceylonensis*)	*An*. *balabacensis*	Detection of parasite DNA in sporozoite-infected salivary gland. Sabah, Malaysian Borneo [68]	Brunei, Indonesia, Malaysian Borneo, and Philippines [79]
	^a^*An*. *cracens* (*An*. *balabacensis balabacensis* by Cheong et al. 1965)	Inoculation of sporozoites into rhesus macaque. Perlis, Peninsular Malaysia [81]	Indonesia, Peninsular Malaysia, and Thailand [79]
	*An*. *dirus*	Detection of parasite DNA in sporozoite-infected salivary glands. Vietnam [66]	Cambodia, Lao PDR, Thailand, and Vietnam [73]
	*An*. *hackeri*	Inoculation of sporozoites into rhesus macaque. Peninsular Malaysia [80]	Malaysian Borneo, Peninsular Malaysia, Philippines, and Thailand [73,79]
	^a^*An*. *introlatus* (*An*. *balabacensis introlatus* by Eyles et al. 1963)	Inoculation of sporozoites into rhesus macaque. Peninsular Malaysia [82]	Indonesia, Peninsular Malaysia, and Thailand [73,79]
*P*. *fieldi*	*An*. *balabacensis* (?)	Detection of parasite DNA in whole mosquito. Sabah, Malaysian Borneo [77]	Brunei, Indonesia, Malaysian Borneo, and Philippines [79]
	*An*. *hackeri*	Inoculation of sporozoites into rhesus macaque [80]	Malaysian Borneo, Peninsular Malaysia, Philippines, and Thailand [73,79]
	^a^*An*. *introlatus* (*An*. *balabacensis introlatus* by Warren and Wharton 1963)	Inoculation of sporozoites into rhesus macaque [80]	Indonesia, Peninsular Malaysia, and Thailand [73,79]
*P*. *inui* (including the variant *shortti*)	*An*. *balabacensis*	Detection of parasite DNA in sporozoite-infected salivary gland. Sabah, Malaysian Borneo [68]	Brunei, Indonesia, Malaysian Borneo, and Philippines [79]
	^a^*An*. *cracens* (*An*. *balabacensis balabacensis* by Cheong et al. 1965)	Inoculation of sporozoites into rhesus macaque. Perlis, Peninsular Malaysia [81]	Indonesia, Peninsular Malaysia, and Thailand [79]
	*An*. *dirus*	Detection of parasite DNA in sporozoite-infected salivary glands. Vietnam [66]	Cambodia, Lao PDR, Thailand, and Vietnam [73]
	*An*. *hackeri*	Inoculation of sporozoites into rhesus macaque [80]	Malaysian Borneo, Peninsular Malaysia, Philippines, and Thailand [73,79]
	^a^*An*. *latens* (*An*. *leucosphyrus* by Wharton et al. 1962)	Inoculation of sporozoites into rhesus macaque. Selangor, Peninsular Malaysia [83]	Indonesia, Malaysian Borneo, Peninsular Malaysia, and Thailand [79]
*P*. *knowlesi*	*An*. *balabacensis*	Detection of parasite DNA in sporozoite-infected salivary gland. Sabah, Malaysian Borneo [68]	Brunei, Indonesia, Malaysian Borneo, and Philippines [79]
	*An*. *cracens*	Detection of parasite DNA in sporozoite-infected salivary gland. Pahang, Peninsular Malaysia [70]	Indonesia, Peninsular Malaysia, and Thailand [79]
	*An*. *dirus*	Detection of parasite DNA in sporozoite-infected salivary gland. Vietnam [42]	Cambodia, Lao PDR, Thailand, and Vietnam [73]
	*An*. introlatus*	Detection of parasite DNA in the oocysts. Selangor, Peninsular Malaysia [71]	Indonesia, Peninsular Malaysia, and Thailand [73,79]
	*An*. *hackeri*	Inoculation of sporozoites into rhesus macaque. Selangor, Peninsular Malaysia [4]	Malaysian Borneo, Peninsular Malaysia, Philippines, and Thailand [73,79]
	*An*. *latens*	Detection of parasite DNA in sporozoite-infected salivary gland. Sarawak, Malaysian Borneo [67]	Indonesia, Malaysian Borneo, Peninsular Malaysia, and Thailand [79]

^a^ Species name revised based on Sallum et al. 2005 [79].

(^?^) The parasite DNA was detected in the whole body (not in saliva or sporozoite form, so it is still questionable).

* Vector had been incriminated based on epidemiological grounds.

Lao PDR, Lao People’s Democratic Republic; SEA, Southeast Asia.

## Challenges and future direction: What’s next?

### Increasing case numbers of knowlesi malaria

With the reduction in human malaria cases, in Southeast Asia, more and more cases of simian malaria, especially due to *P*. *knowlesi*, are detected in humans as seen in Malaysia and Sumatra in Indonesia [23]. One factor could be simply increased awareness and the availability of molecular diagnosis. Besides, increasing exposure of humans to mosquitoes that transmit simian malaria may also be a factor. This is compounded by human encroachment into areas where deforestation and environmental changes are taking place.

However, this hardly explains the continued steep increases in Malaysia over the last 25 years. With reduction of human malaria, control measures may be relaxed, increasing the risk of simian malaria. In addition, diminishing cross-species protective immunity may play a role. *P*. *knowlesi* and *P*. *vivax* share important antigenic properties [84–86]. In vitro, specific antibodies against *P*. *vivax* antigens from animals and malaria patients inhibit erythrocyte invasion by *P*. *knowlesi* [86,87]. Data from neurosyphilis malariotherapy demonstrated that patients previously infected with *P*. *vivax* had lower susceptibility to *P*. *knowlesi* [88].

In addition, are we beginning to see human-to-human transmission occurring? At least in some areas, in Malaysia and Vietnam, the main vectors of simian malaria are also the main vectors of human malaria. While monkey-to-human transmission currently remains the main route of transmission for knowlesi malaria [89], human-to-human transmission could well occur, and may become more likely as prevalence continues to increase.

### Possibilities of other simian malaria infecting humans

As noted above, cases of other simian malaria parasites infecting humans have been detected with increasing frequency over the last 10 years. With reports of *P*. *cynomolgi* in humans [11–15], the results of the study demonstrate the importance of being proactive to prevent outbreaks. Previous studies have indicated that antisera to *P*. *falciparum*, *P*. *malariae* and *P*. *ovale* from humans can cross-react with the *P*. *cynomolgi* antigen [90–92]. Therefore, if *P*. *knowlesi* emerged in humans due to the waning cross-immunity [45], this could occur also for other simian malarias.

As *P*. *inui* and *P*. *cynomolgi* are the most prevalent simian malaria parasites in the macaque hosts and vectors in Southeast Asia, humans are exposed to these parasites by bites of infectious mosquitoes [66]. The vectors of simian malaria are now known to bite both macaques and humans [67,68,70] unlike in the 1960s when *An*. *hackeri* was the only verified vector of simian malaria [4]. As human infections with *P*. *inui* and *P*. *cynomolgi* result in milder infections which do not require hospitalization compared to infection caused by human malaria or *P*. *knowlesi* [93], these infections could go unnoticed as asymptomatic infections or be misdiagnosed. Under these circumstances, those parasites could adapt to humans in the years to come. Indeed, some studies have shown that host switching is common in the evolution of malaria parasites [94,95]. Thus, we believe that the surveillance and investigation of naturally acquired infections with other simian plasmodia should be given high priority.

### Control of simian malarias

While human malaria control strategies mainly rely on insecticide-treated nets or indoor residual spraying and chemotherapy, the control of simian malaria poses a serious challenge, as neither the nonhuman primate reservoir nor the mainly forest-dwelling vectors are much affected by the human-focused interventions. A study in Sabah, Malaysia [96] indicated that mosquito nets have less protective effect against *P*. *knowlesi* infection mainly due to the bionomics of the local vector, *An*. *balabacensis*. New vector control tools like repellents and insecticide treated clothing need to be investigated. Besides, more robust vector studies should be conducted to enable the design of new tools for vector control. Given the importance of environmental change for the epidemiology of these infections, Forest, Agricultural, and Public Health Departments should work together to identify and investigate possible infections thoroughly and seek to curb transmission [74].

Certification of malaria-free status is granted by WHO if the transmission of human malaria in an entire country has been interrupted for at least 3 consecutive years [97]. For Southeast Asian countries aiming to obtain WHO certification of malaria-free status, it would be helpful if WHO could state whether *P*. *knowlesi* is to be considered a human malaria parasite in the context of certification. More generally, we call on WHO to spearhead the recognition of simian malarias in humans as a threat, for example, (but not only) by including simian malaria in the annual World Malaria Reports. This could spur increased surveillance and research to the benefit of the populations at risk and the ultimate goal of malaria eradication.

### Case management of simian malarias

A rapid diagnostic test with high specificity and sensitivity is yet to be developed for *P*. *knowlesi*. In moving forward, it is also crucial to initiate the development of diagnostic kits for both *P*. *inui* and *P*. *cynomolgi*. In a recent case reported in Kelantan, Malaysia [98], a patient infected with *P*. *cynomolgi* was misdiagnosed as *P*. *vivax* through microscopic examinations. When later reexamined molecularly, it was found that the patient was infected with *P*. *cynomolgi*, not *P*. *vivax*. Furthermore, the knowledge of clinicians about simian malarias is insufficient [98]. It is not generally known, for example, that *P*. *cynomolgi* produce hypnozoites [99] and thus requires primaquine treatment.

## Conclusions

Many countries in Southeast Asia are rapidly reducing indigenous human malaria transmission. However, the ongoing increase in *P*. *knowlesi* cases poses a major challenge to malaria control. Besides *P*. *knowlesi*, priority should be given to surveillance and control of other simian *Plasmodium* species, especially *P*. *inui* and *P*. *cynomolgi*, which have the potential to infect human. Simian malaria could emerge and spread as a major public health problem, as the 4 classical human malarias are reduced and as environmental change brings human into increased contact with simian malaria. The early recognition and containment of transmission of these simian malaria among humans should be given high priority.

**Key Learning Points**

- The reduction in human malaria cases may have exposed human populations in some areas of Southeast Asia to a greater risk of being infected by *Plasmodium knowlesi* due to diminishing cross-species immunity.
- Increased number of imported cases of simian malaria in countries outside Southeast Asia shows that simian malaria is now an international concern.
- *Plasmodium cynomolgi* and *Plasmodium inui* are more prevalent in *Anopheles* vectors and macaques hosts in Southeast Asia than *P*. *knowlesi*, raising the possibility of increased natural transmission of these simian malaria parasites to human in future.
- The possible emergence of other simian parasites (besides *P*. *knowlesi*) necessitates rapid diagnostic tests, appropriate treatment regimes, and new control strategies for malaria caused by these parasites.

**Top Five Papers**

1. Grignard L, Shah S, Chua TH, William T, Drakeley CJ, Fornace KM. Natural human infections with *Plasmodium cynomolgi* and other malaria species in an elimination setting in Sabah, Malaysia. J Infect Dis. 2019;220(12):1946–9.
2. Herdiana H, Irnawati I, Coutrier FN, Munthe A, Mardiati M, Yuniarti T, et al. Two clusters of *Plasmodium knowlesi* cases in a malaria elimination area, Sabang Municipality, Aceh, Indonesia. Malar J. 2018;17(1):186.
3. Lee KS, Divis PCSS, Zakaria SK, Matusop A, Julin RA, Conway DJ, et al. *Plasmodium knowlesi*: Reservoir hosts and tracking the emergence in humans and macaques. PLoS Pathog. 2011;7(4):e1002015.
4. Moyes CL, Henry AJ, Golding N, Huang Z, Singh B, Baird JK, et al. Defining the geographical range of the *Plasmodium knowlesi* reservoir. PLoS Negl Trop Dis. 2014;8(3):e2780.
5. Wong ML, Chua TH, Leong CS, Khaw LT, Fornace K, Wan-Sulaiman W-Y, et al. Seasonal and spatial dynamics of the primary vector of *Plasmodium knowlesi* within a major transmission focus in Sabah, Malaysia. PLOS Negl Trop Dis. 2015;9(10):e0004135.

## Supporting information

S1 Table Imported cases of *P*. *knowlesi* malaria among intercontinental travelers. (DOC)
