# Supplementary material for: Plasmodium knowlesi infecting humans in Southeast Asia: What’s next?
Source: PLoS Negl Trop Dis. 2020 Dec 31;14(12):e0008900. doi: 10.1371/journal.pntd.0008900 (PMC7774830; doi:10.1371/journal.pntd.0008900)
Supplement: S1 Table — (DOC) [file pntd.0008900.s001.doc]

**Supporting information**

**S1 Table: Imported cases of *P. knowlesi* malaria among intercontinental travelers.**

| **Year of acquisition** | **Country of presentation** | **Presumed area of acquisition** | **Reference** |
| --- | --- | --- | --- |
| 1965 | USA | Pahang, Peninsular Malaysia | [1] |
| 2005 | Taiwan | Island of Palawan, Philippines | [2] |
| 2006 | Sweden | Sarawak, Malaysia | [3] |
| 2007 | Finland | Rural areas around Kuala Lumpur + South Ipoh + Langkawi, Peninsular Malaysia | [4] |
| 2008 | USA | Island of Palawan, Philippines | [5] |
| 2008/2009 | Spain | Bangkok, Thailand; Banda Aceh + Pulau Weh, Indonesia; Kuala Lumpur, Malaysia; Hanoi, Vietnam | [6] |
| 2009 | Netherlands | Sarawak, Malaysia | [7] |
| 2010 | Australia | Kalimantan, Indonesia | [8] |
| 2010 | New Zealand | Borneo Malaysia | [9] |
| 2010 | France | Ranong and Island of Ko Phayam, Thailand | [10] |
| 2011 | Netherlands | Borneo Malaysia | [11] |
| 2012 | Japan | Temengor Perak, Peninsular Malaysia | [12] |
| 2013 | Germany | Ranong, Thailand | [13] |
| 2013 | Germany | Khoa Sok, Thailand | [14] |
| 2013 | Germany | Island of Mecleod, Myanmar; Island Koh Ra + Khao Lak + Phuket, Thailand | [15] |
| 2014 | Scotland | Borneo Malaysia | [16] |
| NA | Germany | Island of Ko Chang + Krabi, Thailand | [17] |
| NA | Turkey | Myitkyina, Myanmar | [18] |
| 2016 | Sri Lanka | Pulada Johor, Peninsular Malaysia | [19] |
| 2016 | Italy | Island of Palawan + Siquijor + Bohol, Philippines | [20] |
| 2016/2017 | Germany | Chiang Mai + Bangkok + Ranong city + Island of Little Koh Chang, Thailand | [21] |
| 2017/2018* | Japan | Island of Palawan, Philippines | [22] |
| 2018 | Poland | Sumatera, Indonesia | [23] |

*Year not clearly specified

References

1. Chin W, Contacos PG, Coatney GR, Kimball HR. A naturally acquired quotidian-type malaria in man transferable to monkeys. Science. 1965;149(80):865.

2. Kuo MC, Chiang TY, Chan CW, Tsai WS, Ji DD. A case report of simian malaria, *Plasmodium knowlesi*, in a Taiwanese traveller from Palawan island, the Philippines. Taiwan Epidemiol Bull. 2009;25:178–191.

3. Bronner U, Divis PCS, Farnert A, Singh B. Swedish traveller with *Plasmodium knowlesi* malaria after visiting Malaysian Borneo: a case report. Malar J. 2009;8(1):15.

4. Kantele A, Marti H, Felger I, Müller D, Jokiranta TS. Monkey malaria in a European traveler returning from Malaysia. Emerg Infect Dis. 2008;14(9):1434–1436.

5. Ennis J, Teal A, Habura A, Madison-Antenucci S, Keithly J, Arguin P, et al. Simian malaria in a US traveler-New York, 2008. MMWR Morb Mortal Wkly Rep. 2009;58(9):229–232.

6. Tang THT, Salas A, Ali-Tammam M, Del Carmen Martínez M, Lanza M, Arroyo E, et al. First case of detection of *Plasmodium knowlesi* in Spain by real time PCR in a traveller from Southeast Asia. Malar J. 2010;9(1):1-6.

7. van Hellemond JJ, Rutten M, Koelewijn R, Zeeman A-M, Verweij JJ, Wismans PJ, et al. Human *Plasmodium knowlesi* infection detected by rapid diagnostic tests for malaria. Emerg Infect Dis. 2009;15(9): 1478–1480.

8. Figtree M, Lee R, Bain L, Kennedy T, Mackertich S, Urban M, et al. *Plasmodium knowlesi* in human, Indonesian Borneo. Emerg Infect Dis. 2010;16(4):672–674.

9. Hoosen A, Shaw MTM. *Plasmodium knowlesi* in a traveller returning to New Zealand. Travel Med Infect Dis. 2011;9(3):144–148.

10. Berry A, Coustumier A Le, Wilhelm N, Olagnier D, Sire S, Iriart X, et al. Imported *Plasmodium knowlesi* malaria in a French tourist returning from Thailand. Am J Trop Med Hyg. 2011;84(4):535–538.

11. Link L, Bart A, Verhaar N, van Gool T, Pronk M, Scharnhorst V. Molecular detection of *Plasmodium knowlesi* in a Dutch traveler by Real-Time PCR. J Clin Microbiol. 2012;50(7): 2523–2524.

12. Tanizaki R, Ujiie M, Kato Y, Iwagami M, Hashimoto A, Kutsuna S, et al. First case of *Plasmodium knowlesi* infection in a Japanese traveller returning from Malaysia. Malar J. 2013;12(1):128.

13. Ehrhardt J, Trein A, Kremsner P, Frank M. *Plasmodium knowlesi* and HIV co-infection in a German traveller to Thailand. Malar J. 2013;12(1):283.

14. Orth H, Jensen B, Holtfreter M, Kocheril S, Mallach S, MacKenzie C, et al. *Plasmodium knowlesi* infection imported to Germany, January 2013. Euro surveill. 2013;18(40):20603.

15. Seilmaier M, Hartmann W, Beissner M, Fenzl T, Haller C, Guggemos W, et al. Severe *Plasmodium knowlesi* infection with multi-organ failure imported to Germany from Thailand/Myanmar. Malar J. 2014;13(1):422.

16. Cordina CJ, Culleton R, Jones BL, Smith CC, MacConnachie AA, Coyne MJ, et al. *Plasmodium knowlesi*: Clinical presentation and laboratory diagnosis of the first human case in a Scottish traveler. J Travel Med. 2014;21(5):357–360.

17. Kroidl I, Seilmaier M, Berens-Riha N, Bretzel G, Wendtner C, Löscher T. Monkey malaria (*Plasmodium knowlesi* infection) after travelling to Thailand. Dtsch Med Wochenschr. 2015;140(11):815–817.

18. Özbilgin A, Çavuş İ, Yildirim A, Gündüz C. The first monkey malaria in Turkey: A case of *Plasmodium knowlesi*. Mikrobiyol Bul. 2016;50(3):484–490.

19. Ranaweera AD, Danansuriya MN, Pahalagedera K, de AW Gunasekera WM, Dharmawardena P, Mak KW, et al. Diagnostic challenges and case management of the first imported case of *Plasmodium knowlesi* in Sri Lanka. Malar J. 2017;16(1):126.

20. De Canale E, Sgarabotto D, Marini G, Menegotto N, Masiero S, Akkouche W, et al. *Plasmodium knowlesi* malaria in a traveller returning from the Philippines to Italy, 2016. New Microbiol. 2017;40(4):291–294.

21. Froeschl G, Beissner M, Huber K, Bretzel G, Hoelscher M, Rothe C. *Plasmodium knowlesi* infection in a returning German traveller from Thailand: A case report on an emerging malaria pathogen in a popular low-risk travel destination. BMC Infect Dis. 2018;18(1):148.

22. Takaya S, Kano S, Kutsuna S, Suzuki T, Komaki-Yasuda K, Ohmagari N. Case report: *Plasmodium knowlesi* infection with rhabdomyolysis in a Japanese traveler to Palawan, the Philippines. Am J Trop Med Hyg. 2018;99(4):967–969.

23. Nowak SP, PawełZmora, Pielok Ł, Kuszel Ł, Kierzek R, Stefaniak J, et al. Case of *Plasmodium knowlesi* malaria in Poland linked to travel in Southeast Asia. Emerg Infect Dis. 2019;25(9):1772–1773.
